# The potential therapeutic role of extracellular vesicles in critical-size bone defects: Spring of cell-free regenerative medicine is coming

**DOI:** 10.3389/fbioe.2023.1050916

**Published:** 2023-01-17

**Authors:** Fen Liu, Tianyu Sun, Ying An, Leiguo Ming, Yinghui Li, Zhifei Zhou, Fengqing Shang

**Affiliations:** ^1^ Department of Periodontology, Shenzhen Stomatological Hospital (Pingshan), Southern Medical University, Shenzhen, Guangdong, China; ^2^ Department of Periodontology, Stomatological Hospital, Southern Medical University, Guangzhou, Guangdong, China; ^3^ State Key Laboratory of Military Stomatology and National Clinical Research Center for Oral Diseases and Shaanxi Engineering Research Center for Dental Materials and Advanced Manufacture and Department of Periodontology, School of Stomatology, Fourth Military Medical University, Xi’an, Shaanxi, China; ^4^ Department of Research and Development, Shaanxi Zhonghong Institute of Regenerative Medicine, Xi’an, Shaanxi, China; ^5^ Department of Orthodontics, Stomatological Hospital, Hebei Medical University, Shijiazhuang, Hebei, China; ^6^ Department of Stomatology, General Hospital of Tibetan Military Command, Lhasa, Tibet, China; ^7^ Department of Stomatology, Air Force Medical Center, Fourth Military Medical University, Beijing, China

**Keywords:** extracellular vesicles, critical-size bone defects, cell-free therapy, bioactive scaffold, bone tissue regeneration, engineering modification

## Abstract

In recent years, the incidence of critical-size bone defects has significantly increased. Critical-size bone defects seriously affect patients’ motor functions and quality of life and increase the need for additional clinical treatments. Bone tissue engineering (BTE) has made great progress in repairing critical-size bone defects. As one of the main components of bone tissue engineering, stem cell-based therapy is considered a potential effective strategy to regenerate bone tissues. However, there are some disadvantages including phenotypic changes, immune rejection, potential tumorigenicity, low homing efficiency and cell survival rate that restrict its wider clinical applications. Evidence has shown that the positive biological effects of stem cells on tissue repair are largely mediated through paracrine action by nanostructured extracellular vesicles (EVs), which may overcome the limitations of traditional stem cell-based treatments. In addition to stem cell-derived extracellular vesicles, the potential therapeutic roles of nonstem cell-derived extracellular vesicles in critical-size bone defect repair have also attracted attention from scholars in recent years. Currently, the development of extracellular vesicles-mediated cell-free regenerative medicine is still in the preliminary stage, and the specific mechanisms remain elusive. Herein, the authors first review the research progress and possible mechanisms of extracellular vesicles combined with bone tissue engineering scaffolds to promote bone regeneration *via* bioactive molecules. Engineering modified extracellular vesicles is an emerging component of bone tissue engineering and its main progression and clinical applications will be discussed. Finally, future perspectives and challenges of developing extracellular vesicle-based regenerative medicine will be given. This review may provide a theoretical basis for the future development of extracellular vesicle-based biomedicine and provide clinical references for promoting the repair of critical-size bone defects.

## 1 Introduction

Critical-size bone defects refer to the smallest intraosseous wound that cannot heal spontaneously (Falacho et al., 2021). The aetiologies include trauma, tumour and infection (Shang et al., 2020). In today’s rapidly ageing global population, the incidence of this disease has significantly increased. Critical-size bone defects considerably affect patients’ motor function and quality of life and increase the need for additional clinical treatments (Nauth et al., 2018). Currently, the clinical strategies for repairing critical-size bone defects mainly include autologous or allogeneic bone transplantation and synthetic biomaterial transplantation (Yu et al., 2022). Autologous bone transplantation is generally considered the gold standard for repairing critical-size bone defects; however, there are many limitations, including injury to the donor site, limited source, and unpredicted spontaneous resorption and disease transmission risk (Stahl and Yang, 2021). The clinical application of pure allogeneic bone transplantation also has some disadvantages, manifested by immune rejection (Behrend et al., 2016) or unwanted disease transmission (Baldwin et al., 2019).

Bone tissue engineering (BTE), which uses biomaterials with sophisticated biophysical or biochemical features to circumvent the constraints described above, has made great progress in repairing critical-size bone defects. BTE includes three basic components: biological scaffolds, seed cells and bone inductive factors (Roseti et al., 2017). Biological scaffolds are commonly referred to as bioactive materials, which can be combined with mesenchymal stem cells (MSCs) and their secreted cytokines to repair critical-size bone defects (Wu et al., 2021). MSCs are a heterogeneous mesenchymal stem cell subpopulation with the potential for multidirectional differentiation into mesoderm, ectoderm and endoderm lineages (Bianco and Gehron Robey, 2000). Therefore, stem cell therapy is considered to be a potential effective strategy to regenerate bone tissues (Silva et al., 2021). However, stem cell therapy also has some disadvantages that restrict its wider clinical applications, including phenotypic changes, immune rejection, potential tumorigenicity, low homing efficiency and cell survival rate (Sissung and Figg, 2020). These concerns, together with problems caused by storage and transport, further limit the application of MSCs in repairing critical-size bone defects (Nagelkerke et al., 2021). In addition, even after successful transplantation, factors such as donor age, passage times of *in vitro* expansion, culture conditions, cell transplantation procedures and recipient pathological microenvironments also have adverse effects on the survival and biological characteristics of cells (Rezaie et al., 2018). Many studies have attempted to overcome these shortcomings by genetically modifying MSCs and optimizing their culture conditions (García-Sánchez et al., 2019), but key limitations on the biological safety of MSCs still exist.

**FIGURE 1:**
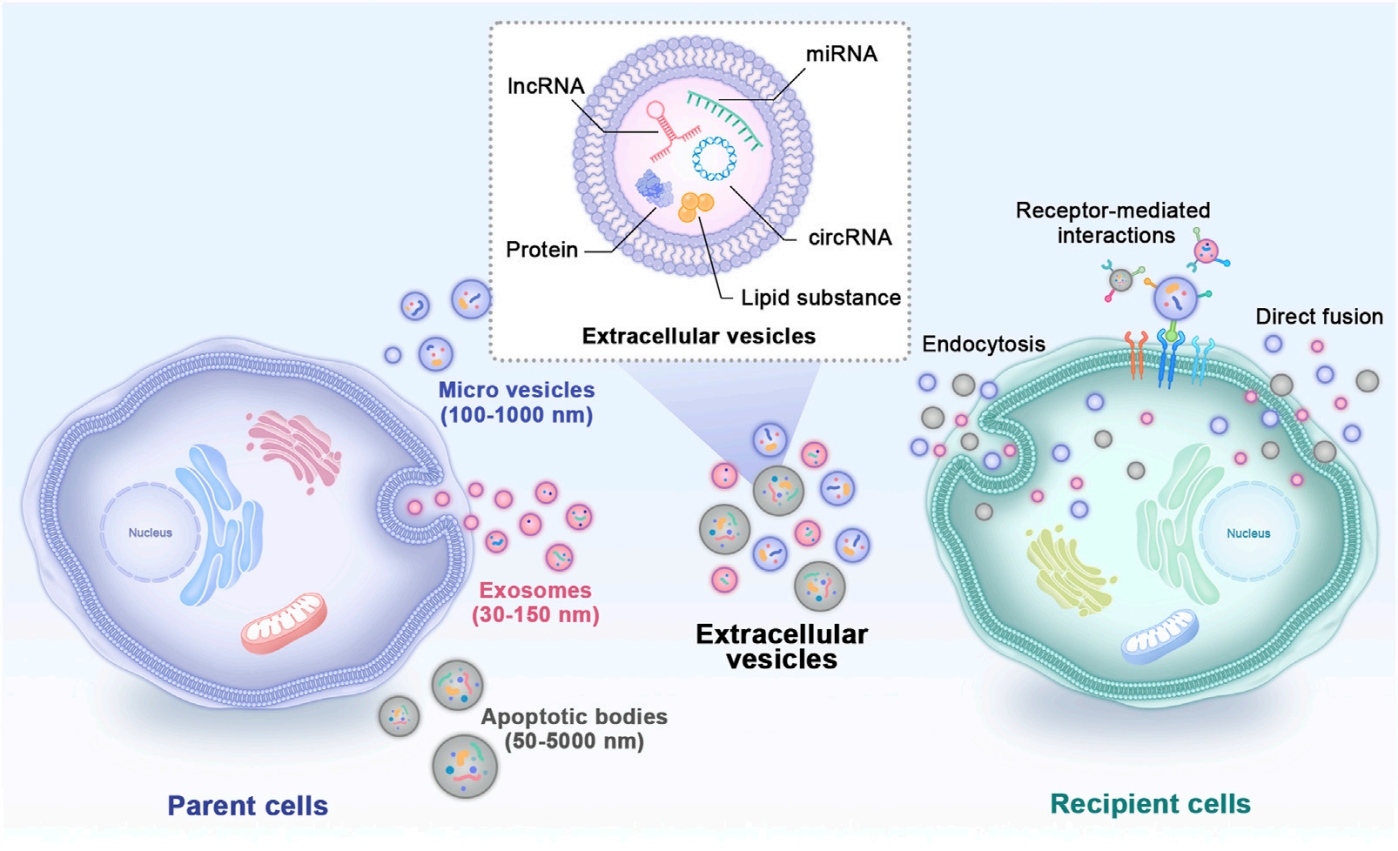
Types and internal cargoes of EVs and the way they act on target cells. EVs mainly include exosomes, microvesicles and apoptotic bodies. EVs carry abundant amounts of proteins, miRNAs, mRNAs, circRNAs and other biological molecules derived from parent cells. They transfer internal cargoes to target cells through ligand‒receptor interactions, endocytosis or direct membrane fusion. EVs: extracellular vesicles.

In recent years, cell-free regenerative medicine for critical-size bone defects has received extensive attention (Kang et al., 2022). Evidence has shown that the positive biological effects of MSCs on tissue repair are largely mediated through paracrine action by extracellular vesicles (EVs) rather than direct differentiation into parenchymal cells to repair or replace damaged tissues (Zhang J. et al., 2016). EVs are double phospholipid bilayer vesicles with nanostructures that bud from the membrane of their parent cells and play vital roles in cell-to-cell communications (Vizoso et al., 2017). Based on different diameters, compositions and sources, EVs mainly include exosomes, microvesicles and apoptotic bodies (Figure 1) (Zhou et al., 2022). Previously, the effects of EVs were mistaken for removing the generated waste of parent cells. Currently, EVs are reputed to be typical representatives of nanotherapeutics carrying abundant nucleic acids, proteins, lipid substances and bioactive small molecules derived from parent cells (Puddu et al., 2010). They transfer internal cargoes to target cells through ligand‒receptor interactions, endocytosis or direct membrane fusion to exert regulatory effects (Li Q. C. et al., 2022). Studies have shown that EVs obtained from MSCs have more ideal biocompatibility, effective cell interaction characteristics, exogenous cargo delivery and the ability to target specific tissues (van der Koog et al., 2022), and most importantly, they overcome the limitations of traditional MSC treatment (Malekpour et al., 2022). In recent years, in studies of bone regeneration and homeostasis, EVs have also become remarkable hotspots (Zara et al., 2011). EVs loaded with bioactive materials can significantly promote osteogenesis, angiogenesis and inflammation regulation, thus effectively repairing critical-size bone defects (Liang et al., 2022). Because EV subtypes do not have well-recognized specific markers, the International Society for Extracellular Vesicles recommends using “small EVs” for EV subtypes (Théry et al., 2018). However, most studies used the term “exosomes” in their experiments. In this review, to respect the cited literature, we reserve to use the term “exosomes” when the author gave a specific definition in the article. We use the generic term “EVs” when they were not clearly noted.

Although considerable research on EVs has focused on promoting the repair of critical-size bone defects, their development is still in the preliminary stage, and the specific mechanisms remain elusive. What is more, for critical-size bone defects, application of EVs alone could not fill defect spaces and guide bioactive molecules to repair and reconstruct the bone defect. Thus, some bioactive scaffolds are needed in EVs-based BTE application. Herein, the authors first review the research progress and possible mechanisms of EVs combined with BTE scaffolds to promote bone regeneration *via* bioactive molecules. Engineering modified EVs is an emerging component of BTE. Their main progress and opportunities will then be discussed. Finally, future perspectives and challenges of the developing EV-based regenerative medicine are given. Thus, this review may provide a theoretical basis for the future development of the EV-based biomedicine and provide clinical references for promoting the repair of critical-size bone defects.

## 2 Potential therapeutic applications of extracellular vesicles in bone repair

Tissue regenerative medicine aims to enhance the positive effects of stem cells delivered *in vivo*. To date, multiple studies have demonstrated that very few transplanted cells can be tracked *in vivo* after 4–8 weeks, suggesting that their regenerative ability is more likely to be associated with the indirect mechanisms of seed cells, including cytokine paracrine, immunoregulation, and signal transduction (Caplan, 2019). Based on that, researchers used cell aggregates with abundant extracellular matrix to replace dispersed stem cells, and the obtained results showed promising new bone formation in critical-size bone defects in osteoporosis rats (Shang et al., 2014). Although cell aggregates showed better results in preclinical experiments, limitations of cell-based therapy still rendered it taking a step from the research stage to clinical applications. Stem cell-based regenerative medicine has some disadvantages. These limitations, together with problems caused by storage and transport, further limit the application of MSCs in repairing critical-size bone defects (Sissung and Figg, 2020). EVs could overcome these shortages of stem cells while maintain similar functions in regenerating critical-size bone defects. Besides, effects of stem cells in BTE largely dependent on their paracrine action. Direct use of EVs may lead to more stable and predicable results. These are all reasons for applying EVs in cell-free regenerative medicine. In a femur fracture animal model without EV secretion, delayed bone healing was observed, while the healing situation was improved by injecting additional EVs (Furuta et al., 2016). Moreover, the application of MSC-derived EVs also shortened the recovery time, suggesting that EVs promote bone tissue regeneration. This finding highlighted the perspective of EV-mediated bone regenerative medicine (Gunawardena et al., 2019). Although the study of MSC-derived EVs in regenerative medicine is just at the outset, preclinical tests have shown more positive results and fewer adverse effects compared to applications of MSCs.

### 2.1 Parent cells of extracellular vesicles

EVs were originally observed in reticulocytes during their maturation and were treated as a way to remove waste transferrin receptors (Harding et al., 1983). Afterwards, the phenomenon of EV secretion was found not only in most human fluids but also in a variety of cell types (Johnstone et al., 1987). EVs take chemical or genetic materials to pass messages for cell‒cell communication. Thus, their function is more than that of superfluous membrane vesicles. The possible therapeutic roles of takeover stem cells are being further explored. Mounting evidence indicates that EVs can facilitate cell differentiation and viability and are actively involved in bone homeostasis through cargoes containing microRNAs (miRNAs), proteins and so on.

#### 2.1.1 Mesenchymal stem cells-derived extracellular vesicles

During bone remodelling, the interaction between osteoblasts and osteoclasts is necessary to replace mechanically weak fibrous bone with mechanically strong lamellar bone. Takeuchi et al. (2019) confirmed that MSC-derived EVs manifested the same effects in critical-size bone defect repair with a complete MSC secretome, and both groups had almost half of the defect site covered with new bone tissues after 4 weeks of treatment by promoting angiogenesis and osteogenesis. MSC-derived exosomes, which are the most active vesicles from MSCs (Phan et al., 2018), have been identified as important messengers (Lai et al., 2010) and play a more vital immunoregulatory role than the parent cells (Del Fattore et al., 2015). It has been demonstrated that MSC-derived EVs can accelerate every step of bone defect repair, contributing to bone rehabilitation through immunoregulation. In the regenerative treatment of critical-size bone defects, MSC-derived EVs can stimulate the proliferation and angiogenesis of osteoblasts by delivering endogenous cargoes and inhibit osteoclast maturation (Qin et al., 2016a). Recent studies supported the view that MSC-derived EVs had comparable therapeutic properties compared with their parent cells (Kou et al., 2018). The expression of the protein spectrum showed that 1,927 of a total of 6,342 proteins were expressed in both MSCs and MSC-derived EVs (Anderson et al., 2016). Hence, EVs secreted by different MSCs may inherit matrilineal characteristics in bone regeneration and repair *via* similar mechanisms to parent cells (Tan et al., 2021). Therefore, selecting an applicable source of parent cells is extremely important for ideal applications of EVs in hard tissue regeneration for critical-size bone defects (Behera and Tyagi, 2018). The commonly applied parent MSCs of EVs mainly include bone marrow mesenchymal stem cells (BMMSCs) (Qin et al., 2016b), adipose-derived stem cells (ADSCs) (Li et al., 2018), umbilical cord mesenchymal stem cells (UCMSCs) (Zhang Y. et al., 2021), MSCs derived from induced pluripotent stem cells (iPS-MSCs) (Fu et al., 2012) and stem cells from human exfoliated deciduous teeth (SHEDs) (Wang M. et al., 2020).

**BMMSCs** are a major type of MSCs. Their bioactive molecules are now extensively investigated for better applications. Proteomic analyses indicated that as many as 1,533 proteins were involved in various biological actions (Baberg et al., 2019). Preclinical studies have also shown that BMMSC-derived EVs exerted favourable effects in promoting osteogenesis and angiogenesis of osteoblast precursor cells at the injury sites in a critical-size bone defect model, thus forming ideal bone mineralization (Zhang et al., 2022). Martins et al. (2016) discovered that hBMMSC-derived exosomes could promote osteogenic differentiation, as verified by early alkaline phosphatase (ALP) activation and bone morphogenetic protein 2 (BMP2) upregulation.

**ADSCs** are regarded as one of the most applicable cell sources for EVs because they can be more easily obtained and are widely distributed in the human body (Parker and Katz, 2006). In addition, these cells rapidly proliferate *in vitro* and retain stem cell phenotypes together with less susceptibility to ageing (Mirsaidi et al., 2012). These advantages make them favourable parent cells for increasing EV isolation (McIntosh et al., 2006). Although the osteogenic capacity of ADSCs is still controversial compared with BMMSCs, hADSC-derived exosomes have been proven to exhibit the capabilities of enhancing the proliferation, migration and osteogenic differentiation of hBMMSCs *ex vivo* and accelerating bone regeneration at critical-size bone defect sites *in vivo* (Liu A. et al., 2021).

**UCMSCs** Umbilical cords are cheap and exhaustless stem cell sources (Wang et al., 2004). hUCMSCs are primitive MSC subpopulations obtained from human postnatal waste tissues (Hendijani et al., 2014). Their collection process requires no invasive operation and has no ethical or moral issues like human embryonic stem cells (Zhao et al., 2010). Compared with other MSCs, hUCMSCs exhibit higher pluripotency (Wang et al., 2015), less immunorejection *in vivo* and no tumorigenic risk peculiarities (Can and Karahuseyinoglu, 2007). Instead of directly inducing osteogenic and chondrogenic differentiation, hUCMSCs improved bone regeneration by indirectly inducing angiogenesis. To date, hUCMSCs have shown significant clinical potential and have also attracted extensive attention from scholars in the bone regeneration field (Wang L. et al., 2020). In an animal model of critical-size bone defects of rat femurs, exosomes derived from hUCMSCs promoted angiogenesis at the injury site through hypoxia inducible factor-1α (HIF-1α) to achieve bone repair (Zhang et al., 2019).

**iPS-MSCs** Similar to hUCMSCs, there are neither immunorejection nor ethical problems in the clinical applications of iPSCs. However, some scholars have pointed out that using iPSCs may bring about tumorigenic risks (Jiang et al., 2016). Compared with iPSCs, iPS-MSCs can maintain their self-renewal capacity even after forty passages and have no tumorigenic risk (Lian et al., 2010). In treating bone defects, applications of iPS-MSCs have shown promising prospects in promoting bone repair and regeneration due to their strong proliferation and immunoregulation abilities (Fu et al., 2012). Because EVs from iPS-MSCs have nearly the same biological characteristics as the parent cell, iPS-MSCs have attracted increasing attention as a cell source to promote bone regeneration by using EVs (Zhang J. et al., 2016). *In vivo* studies reported that hiPS-MSC-derived exosomes enhanced osteogenesis in ovariectomized rats with critical-size calvarial defects (Qi et al., 2016). Moreover, iPS-MSC-derived exosomes may play a certain role in bone defect repair by promoting angiogenesis, as previous research has already demonstrated that they have significant therapeutic effects in treating ischaemic diseases (Hu et al., 2015).

**SHEDs** Deciduous teeth are the only organ that are replaceable and exfoliate naturally from the human body. Additionally, there are no ethical issues when harvesting stem cells from them, which makes these cells attractive for clinical therapy. SHEDs are not mature MSCs and have multidirectional differentiation potentials (Martinez Saez et al., 2016). Compared with BMMSCs, SHEDs showed increased proliferation ability because they secreted more growth factors, including fibroblast growth factor 2 and transforming growth factor-β2 (TGF-β2) (Nakamura et al., 2009). Studies have also demonstrated that SHED-derived exosomes combined with tricalcium phosphate (TCP) could improve alveolar bone repair by inducing angiogenesis and osteogenesis (Wu et al., 2019).

#### 2.1.2 Non-mesenchymal stem cells-derived extracellular vesicles

MSCs differentiating into osteoblasts participate not only in endochondral ossification but also in the formation of intramembranous bones. These two procedures are essential for bone formation. In addition to the participation of MSCs, new bone formation and regeneration also require the functions of osteoblasts, osteoclasts, and chondrocytes. In particular, endothelial cells play a part in osteoblast maturation and angiogenesis (Qin et al., 2016a). A favourable microenvironment is essential for ideal osteogenesis and angiogenesis. It is well-known that immunoregulatory-related cells are of critical importance in maintaining an appropriate microenvironment for new bone and vascular formation. Thus, the potential therapeutic roles of non-MSC-derived EVs in critical-size bone defect repair have also attracted attention from scholars in recent years.

**Osteoblasts** are differentiated from BMMSCs. They synthesize and mineralize the bone matrix by releasing collagen and glycoprotein. Osteoblast-derived exosomes were confirmed to regulate Wnt and calcium signalling pathways and miRNAs to induce osteogenic differentiation of BMMSCs (Cui et al., 2016). Osteo-related miRNAs (miR-1192, miR-680 and miR-302a) were expressed in preosteoblast MC3T3-E1-derived exosomes, and the miRNAs in the target cells were also altered to promote their differentiation into osteoblasts (Cui et al., 2016). The effects of these exosomes have even been demonstrated to surpass the original extracellular matrix of parent cells in inducing the directional differentiation of stromal cells (Narayanan et al., 2018). In one study, the comprehensive analysis of EVs showed that the proteome profile changed continuously at different stages of osteoblast mineralization. At the later stage of mineralization, proteins with specific functions in promoting angiogenesis and bone development are enriched in EVs (Davies et al., 2019). A previous study confirmed that osteoblast-derived EVs could regulate osteoclast differentiation through the receptor activator of nuclear factor-κB ligand (RANKL)-RANK signalling pathway. Live cell imaging in the transgenic zebrafish fracture healing model also found internalization of osteoblast-derived EVs within osteoclasts (Kobayashi-Sun et al., 2020).

**Osteocytes** are terminally differentiated cells, occupying the largest proportion of cells that constitute bone tissues. Although soluble factors and signalling molecules secreted from osteocytes are considered to play key roles in the maintenance of bone homeostasis, current studies on EVs derived from osteocytes are very limited. It was confirmed that osteocyte-derived exosomes contained miRNAs that contributed to bone remodelling (Sato et al., 2017). Osteocytes are sensitive to mechanical stress and respond to external stimuli by regulating the internal cargoes of their exosomes. EVs derived from osteocytes can promote osteogenic differentiation when exposed to mechanical stress. RNA sequencing results suggested that the possible mechanisms were to upregulate miR-181b-5p, thereby inhibiting phosphatase and tensin homologue and thus activating the protein kinase B signalling pathway (Lv et al., 2020). In another similar study, osteocytes subjected to fluid shear stress were able to recruit more stromal progenitor cells through specific EVs, thus promoting local bone remodelling (Eichholz et al., 2020). Of note, the processes of repairing critical-size bone defects by osteocyte-derived EVs are negatively regulated by muscle. Myostatin secreted by muscle can upregulate the expression of miR-218 in exosomes derived from osteocytes, thereby blocking runt-related transcription factor 2 (Runx2) and Wnt signalling pathways and ultimately inhibiting the osteogenic differentiation of BMMSCs (Qin et al., 2017).

**Endothelial cells** Angiogenesis plays a key role in bone metabolism, in which endothelial cells comprise a single layer of the inner wall of blood vessels. The endothelial cells carry circulating blood macromolecules for the surrounding cells to guarantee their metabolism. Exosomes derived from endothelial cells highly directionally target bone tissues, inhibiting the development and activity of osteoclasts (Li X. et al., 2016). In a study of distraction osteogenesis, endothelial progenitor cell-derived exosomes promoted angiogenesis by upregulating miR-126, thereby accelerating the formation of new bones at the defect site (Jia et al., 2019). Another study also found that endothelial progenitor cell-derived EVs could directly act on BMMSCs to promote their osteogenic differentiation *in vitro* (Qin and Zhang, 2017).

**Immune cells** in the microenvironment of critical-size bone defects specifically activate osteoblasts or inhibit osteoclasts through paracrine action. EVs derived from immune cells trigger an immune response by presenting certain antigens. Dendritic cell (DC)-derived EVs were demonstrated to have a good osteogenic inductive effect (Cao et al., 2021), and the possible mechanisms were to regulate the Hippo signalling pathway and induce bone regeneration through exosomal miR-335 *via* LATS1 signalling (Cao et al., 2021). DC-derived exosomes also contain immunoregulatory cargoes TGF-β1 and interleukin (IL)-10, which enhance the recruitment of regulatory T-cell in inflammatory responses and ultimately inhibit bone loss caused by osteoclasts (Elashiry et al., 2020). Active DC-derived EVs may bring about the activation of humoral immune responses and CD4^+^/CD8^+^ T-cell, while immature DC-derived EVs were more likely to decrease systematic inflammation (Lindenbergh and Stoorvogel, 2018). In addition to DCs, exosomes from non-polarized M0, polarized M1 (proinflammatory phenotype) and M2 (anti-inflammatory phenotype) macrophages could specifically target BMMSCs to promote their directional osteogenic differentiation (Xia et al., 2020). Macrophage-derived exosomes can also regulate the gene expression of salt-inducible kinase 2/3 through miR-5106, thereby inducing osteoblast differentiation (Xiong et al., 2020). Effective regulation of the cargoes of EVs derived from immune cells will help scholars better understand the crosstalk between different cells in the microenvironment of critical-size bone defects in the future.

### 2.2 Applications of extracellular vesicles-loaded bioactive materials in bone tissue engineering

EV-mediated bone regeneration strategies have attracted increasing attention in the treatment of critical-size bone defects in recent years (Fernandez-Yague et al., 2015). Accumulating studies have shown that EVs alone or loaded in different scaffolds, such as hydrogels, can significantly promote local bone regeneration. Scaffold materials are required to fill defect spaces and guide bioactive molecules, such as EVs, to exert certain effects on bone defect repair and reconstruction, especially critical-size bone defects. EV-loaded scaffold materials have already achieved gratifying results in repairing animal models of critical-size bone defects. It effectively stimulates bone regeneration in critical-size skull defects (Qin et al., 2016b), promotes cartilage repair and subchondral bone regeneration in osteochondral defects (Zhang S. et al., 2016), and enhances angiogenesis in repairing femoral defects (Liu et al., 2017). In mouse animal models, EV-loaded scaffolds also showed a more ideal ability to promote bone regeneration and angiogenesis compared with the application of scaffold materials alone, suggesting that scaffolds rich in EVs are an attractive treatment alternative for repairing critical-size bone defects (Xie et al., 2017). Applications of different types of bioactive materials loaded with EVs in BTE are summarized in Table 1.

**TABLE 1:** Applications and possible mechanisms of different EVs-loaded bioactive scaffold materials in BTE.

Bioactive scaffold materials	Parent cells of loaded EVs	Nanovesicles	Applications and possible mechanisms	References
Hydrogels				
Hydrogel	BMMSCs	EVs	Stimulating bone growth in critical-sized calvarial bone defects through miR-196a by regulating osteoblastic differentiation	Qin et al. (2016b)
Hydrogel	hADSCs	Exosomes	Enhancing the bone regenerative capacity in calvarial defect by miR-375	Chen et al. (2017)
Injectable hydrogel	hUVECs	Exosomes	Promoting callus formation and fracture healing at the early over-active inflammation phase through inhibiting T-cell	Lin et al. (2021)
Alginate-Arg-Gly-Asp modified hydrogels	hMSCs	EVs	Sustained delivery of osteoinductive functional engineered EVs in calvarial defects	Huang et al. (2021)
Sulfur alcohol modified hyaluronic acid-heparin hydrogel	BMMSCs	EVs	Improving bone formation in the critical-size calvarial defects by regulating multiple signaling pathways through miRNA-196a	Qin et al. (2016b)
Extracellular matrix-mimic hydrogel	BMMSCs	Exosomes	Promoting the anabolism of chondrocytes by inhibiting inflammation and promoting growth plate injury repair through extracellular matrix remodeling	Guan et al. (2021)
Chitosan/β-glycerophosphate hydrogel	BMMSCs	EVs	Promoting angiogenesis in critical-sized rat calvarial defects by miR-21 targeting sprouty homolog 2	Wu et al. (2022)
Hyaluronic acid hydrogel	hUCMSCs	Secretion factors	Initiating osteogenesis of BMMSCs and promoting calvarial bone defect repair	Wang et al. (2015)
Hyaluronic acid-alginate hydrogel	hUCMSCs	Exosomes	Enhancing bone regeneration through promoting angiogenesis in critical-sized calvarial defects	Yang et al. (2020b)
Polyethylene glycol/DNA hybrid hydrogel	Stem cells from apical papilla	Exosomes	Promoting vascularized bone regeneration in the mandibular bone defect through highly expressed miRNA-126-5p and miRNA-150-5p	Jing et al. (2022)
Bioceramics				
β-TCP	hBMMSCs	Secretome	Improving MSCs differentiation capacity and reducing cell senescence by miR-10a	Katagiri et al. (2017)
β-TCP	hiPS-MSCs	Exosomes	Promoting bone regeneration in critical-sized calvarial defects by enhancing angiogenesis and the osteoinductivity of β-TCP through activating the PI3K/Akt signaling pathway	Qi et al. (2016) Zhang et al. (2016a)
β-TCP	SHEDs	Exosomes	Promoting neovascularization and new bone formation, through the adenosine 5′-monophosphate-activated protein kinase signaling pathway	Wu et al. (2019)
Mesoporous bioactive glass scaffold	rBMMSCs, rADSCs	Exosomes	Enhancing bone forming and inducing rapid initiation of bone regeneration by let-7a-5p, let-7c-5p, miR-328a-5p and miR-31a-5p targeting activin A receptor 1/2b and regulating Smad1/5/9 phosphorylation in cranial defect	Liu et al. (2021a)
Polylactic acid-calcium silicates-dicalcium phosphate dihydrate	ADSCs	EVs	Enhancing regenerative bone healing by stimulating the osteogenic commitment of hADSCs	Gandolfi et al. (2020)
Metals				
Ti6Al4V scaffolds	Schwann cells	Exosomes	Promoting the migration, proliferation and differentiation of BMMSCs in bone repair	Wu et al. (2020)
Titanium nanotubes	Macrophages	Exosomes	Activating autophagy during osteogenic differentiation	Wei et al. (2019a)
3D-printed titanium alloy scaffolds	hMSCs	Exosomes	Inducing osteogenic differentiation by up-regulating osteogenic miRNAs or down-regulating anti-osteogenic miRNAs to activate the PI3K and MAPK signaling pathways	Zhai et al. (2020)
Zinc	Nitric oxide synthase-1 positive cells	Matrix vesicles	Increasing the ALP activity of osteoblasts	Kawakubo et al. (2011)
Strontium-substituted calcium silicate	BMMSCs	Exosomes	Promoting angiogenesis of hUVECs through elevating miR-146a and inhibiting Smad4 proteins to accelerate developmental vascularization along with the neovascularization and bone regeneration in distal femur defects	Liu et al. (2021b)
Polymers				
Polycaprolactone scaffold	MSCs	Exosomes	Reducing the inflammation stimulated by inflammatory macrophages and further accelerating osteogenic differentiation of MSCs	Wang et al. (2020d)
Tannic acid modified sulfonated polyetheretherketone	BMMSCs	Exosomes	Exerting osteoimmunomodulation effect to promote osteogenesis through promoting macrophage M2 polarization *via* the NF-κB pathway	Fan et al. (2021)
Polydopamine-coating PLGA scaffolds	hADSCs	Exosomes	Promoting bone regeneration in critical-sized calvarial defects	Li et al. (2018)
PLGA-polyethyleneglycol-PLGA	human dental pulp stem cells	Exosomes	Providing pro-mineralization cues to drive local stem/progenitor cells towards osteogenic differentiation in critical-size calvarial bone defect	Swanson et al. (2020)
Others				
Hyaluronic acid	hMSCs	Exosomes	Promoting functional cartilage and subchondral bone repair	Zhang et al. (2022)
Type I collagen, fibronectin	MSCs	Exosomes	Promoting differentiation of MSCs	Narayanan et al. (2016)
Decalcified bone matrix	MSCs	EVs	Enhancing bone regeneration though promoting vascularization	Xie et al. (2017)

ADSCs: adipose derived stem cells; ALP: alkaline phosphatase; BMMSCs: bone marrow mesenchymal stem cells; β-TCP: β-tricalcium phosphate; BTE: bone tissue engineering; EVs: extracellular vesicles; iPS-MSCs: MSCs, derive from induced pluripotent stem cells; MAPK: mitogen-activated protein kinase; MSCs: mesenchymal stem cells; NF-κB: nuclear factor-κB; PI3K: phosphoinositide 3-kinase; PLGA: poly lactic-co-glycolic acid; SHEDs: stem cells from human exfoliated deciduous teeth; Smad: mothers against decapentaplegic homolog; UCMSCs: umbilical cord mesenchymal stem cells; UVECs: umbilical vein endothelial cells.

#### 2.2.1 Applications of extracellular vesicles-loaded hydrogels

As mentioned above, EVs are an ideal seed cell substitute in BTE. In preclinical or clinical applications, it can avert the safety and ethical problems caused by cell-based therapy, and it is also an effective bone inductive substance. However, there are also many limitations in the application of EVs alone in critical-size bone defect repair. The typical one is that due to the clearance of the reticuloendothelial system, EVs are quickly lost *in vivo,* and therefore, it is difficult to reach an effective therapeutic concentration locally. Thus, clinical applications require appropriate scaffold materials to ensure sustained release of EVs and maintain a local effective concentration of EVs (Riau et al., 2019).

Hydrogels with suitable biocompatibility and controlled release kinetics have been regarded as a preferred carrier material for delivering EVs to bone defect sites (Yan et al., 2020). Subcutaneous implantation of type I collagen hydrogel loaded with hBMMSC-derived exosomes showed better regenerative potential through the upregulation of Runx2 and osterix (Narayanan et al., 2016). EVs loaded in the hydrogel could also improve the bioactivity of this material, leading to the acceleration of human umbilical vein endothelial cell (UVEC) growth and differentiation and finally promoting bone regeneration at critical-size bone defect sites. Therapeutic applications of MSC-derived EVs loaded in hydrogels for treating critical-size calvarial defects have shown significant increases in bone volume, bone mineral density, and newly formed bone area (Qi et al., 2016). It was demonstrated that after being loaded into the hydrogels, EVs could be slowly released in a controlled manner. In addition, hydrogel biomaterials can be fully absorbed at defect sites while promoting osteogenic induction and enhancing bone remodelling (Yang S. et al., 2020). Recently, scholars have focused on constructing novel synthetic hydrogel composites to enhance their function as EV carriers to promote bone regeneration. Adding hydroxyapatite to hyaluronic acid and alginate, Yang S. et al. (2020) produced an injectable hydrogel, which could effectively promote osteoblast differentiation at the critical-size bone defect site after loading MSC exosomes. Another hotspot is how to optimize the controlled release of EVs loaded into hydrogels. Huang et al. (2021) generated engineered modified EVs and loaded them into photocrosslinked sodium alginate hydrogels. It was found that the release process of EVs could be sustained for as long as 7 days, and the biological functions of the loaded EVs did not change during the whole research period. Significant bone regeneration could be observed when applying this system to repair critical-size skull defects.

#### 2.2.2 Applications of extracellular vesicles-loaded bioceramics

In the traditional MSC-based BTE used to treat bone defects, cells need to be directly injected into the target sites. After injection, the multidirectional differentiation potential of MSCs helps to regenerate bone tissues. However, because the injected MSCs lack mechanical support and cannot withstand local pressure, this method is generally not applicable, especially for critical-size bone defect repair (Korhonen and Jurvelin, 2010). To overcome this limitation, bioceramics have become a preferred scaffold material for transplanting cells or cytokines. Porous bioceramics have ideal mechanical strength, biocompatibility and biodegradability (Huang X. et al., 2020). Currently, there are absorbable bioceramics, such as β-TCP, and non-absorbable bioceramics, such as zirconia and alumina, applied in the clinic. Some bioceramics even have biological activities, such as hydroxyapatite glass ceramics. EVs derived from MSCs have similar biological characteristics to MSCs. Therefore, many scholars have loaded them into bioceramic scaffolds to improve the bioactivities of this material. It was found that loading EVs from BMMSCs into hydroxyapatite/TCP bioceramics could better induce new bone formation (Wang et al., 2015). Osteoconductive β-TCP could release hiPSC-MSC-derived exosomes in a controlled manner *in vitro*, thus stimulating the proliferation, migration and osteogenic differentiation of homing hBMMSCs in the injury sites to promote new bone formation. Application of this delivery system loaded with EVs in critical-size calvaria defects showed increased new bone formation through upregulating the expression of osteocalcin (OCN) and osteopontin along with increased levels of the vascular marker CD31 both in healthy (Zhang J. et al., 2016) and osteoporotic rats (Qi et al., 2016). Currently, scholars are also trying to better improve the survival microenvironment of EVs by adding minerals that can enhance the bioactivity of bioceramic materials so that EVs released from them can maintain favourable biological functions to play a bridge role in intercellular communications and promote osteogenic differentiation of stromal cells (Gandolfi et al., 2020). In a recent study, scholars used strontium to replace calcium silicate in the original scaffold materials and found that EVs loaded into new bioceramics could better exert osteogenic differentiation and angiogenesis potentials in zebrafish and rat femoral bone defect models, resulting in ideal regeneration of bone tissues and blood vessels at the defect sites. The possible mechanisms may be that composition changes upregulate the expression of miR-146a in EVs, which in turn inhibits the protein expression of mothers against decapentaplegic homologue (Smad) 4 and NF2 (Liu L. et al., 2021). In addition to modifying the compositions of bioceramics, decreasing the porous diameters would also create a better survival microenvironment for EVs. Micrometer-scale porous (.5–2 μm) graded mesoporous bioactive glass with osteogenesis, angiogenesis, and antimicrobial activities may provide BMMSC-derived exosomes with a larger surface area to meet the bone regeneration demand (Liu A. et al., 2021). In addition to optimizing the composition and construction of bioceramic materials, similar to hydrogels, scholars are also further exploring and improving the release kinetics of EVs loaded into them *in vivo*.

#### 2.2.3 Applications of extracellular vesicles-loaded polymers

Synthetic biodegradable polymers are considered effective delivery carriers of EVs because of their unique advantages, such as adjustable release kinetics. Exosomes from ADSCs were loaded into polylactic-co-glycolic acid (PLGA) scaffolds with a polydopamine coating. *In vitro*, the sustained release of exosomes could be observed, while *in vivo*, it would promote bone regeneration of critical-size bone defects by enhancing homing of BMMSCs to the defect sites (Li et al., 2018). Scholars have also explored copolymer systems, such as PLGA-polyethylene glycol triblock, as a loading platform for the controlled release of EVs and found that its ability to induce osteogenesis is superior to that of exogenous direct delivery of EVs (Swanson et al., 2020). Polycaprolactone is another biomaterial with specificity for inducing bone regeneration. It has a degradable property. Loaded with EVs derived from BMMSCs, polycaprolactone could effectively repair bone defects *in vivo* (Wang X. et al., 2020). Polymer composites have the same elastic modulus as bone tissues, but their biological activity is weak compared with certain types of hydrogels or bioceramics. Therefore, the ability to repair critical-size bone defects *in vivo* could be further improved after loading with EVs.

#### 2.2.4 Applications of extracellular vesicles-loaded metals

As the most traditional bone repair material, metal has good mechanical properties (Zhao et al., 2021). Titanium and its alloys are the most widely used metals in BTE. They have ideal biocompatibility, including non-toxicity, optimal porosity suitable for cell migration and proliferation, high mechanical strength and corrosion resistance. In addition, they are the only metals that have been proven to have osseointegration characteristics (Briguglio et al., 2019). Similar to polymers, many studies that used metals as scaffold materials for EVs have focused on improving their biological functions. Some scholars generated titanium scaffolds by selective laser sintering and 3D printing and then decorated them with EVs derived from hMSCs with preosteogenic differentiation for 4–20 days (Liang et al., 2020). Meanwhile, the adsorption of EVs on the scaffold material was enhanced by coating the scaffold surface with positively charged polylysine to mediate charge interaction. RNA sequencing results suggested that these treatments could simultaneously upregulate specific osteogenic miRNAs and downregulate anti-osteogenic miRNAs of loaded EVs to activate mitogen-activated protein kinase (MAPK) and phosphoinositide 3-kinase (PI3K)/Akt signals and finally obtained good bone induction effects in a rat radial bone defect model (Zhai et al., 2020). In addition, researchers have also tried to produce titanium alloys with porous properties and matching the elastic modulus of bone by 3D printing. After loading Schwann cell-derived exosomes, it was found that the modified titanium alloy had a better ability to repair critical-size bone defects (Wu et al., 2020). In another attempt to improve the function of implanted titanium, researchers loaded BMP2 prestimulated macrophage-derived exosomes into titanium nanotubes and found that surrounding BMMSCs had significantly enhanced osteogenic differentiation ability compared with applying scaffold materials alone (Wei F. et al., 2019). Polyetheretherketone, which has solid mechanical strength and favourable biocompatibility, such as transmissivity and anti-chemical corrosion, is now regarded as an alternative metal scaffold in BTE. After etching with concentrated sulfuric acid, the surface of polyethe retherketone showed a 3D porous structure, which was ideal for the loading and distribution of exosomes. Thus, this system was better at promoting osseointegration by regulating inflammatory responses. Detailed mechanisms include blocking nuclear factor-κB (NF-κB) signalling and enhancing macrophage M2 polarization, and these two processes are reversible (Wei R. et al., 2019). Moreover, tannic acid coated with polyphenol systems has been developed to form reversible hydrogen connections between EVs and scaffold materials, allowing the long-term release of EVs to be realized. The sustained release of exosomes would formulate an immune microenvironment ideal for bone regeneration (Liu and Xiong, 2021c).

In summary, EVs-based cell free regenerative medicine is playing a more and more important role in critical-size bone defect repair. MSC-derived EVs have similar effects to their parental cells while non-MSC-derived EVs have their own advantages in regulating microenvironment. In repairing critical-size bone defects, both MSC and non-MSC-derived EVs need the support of bioactive scaffold materials. These scaffold materials would not only maintain the shape of critical-size bone defect, but also help EVs to better exert their effects through different mechanisms. In future, according to different characteristics of diverse bioactive scaffold materials, more studies are needed to provide references for choosing more favorable EVs under different pathological or regenerative regulatory microenvironments.

## 3 Mechanisms of extracellular vesicles on critical-size bone defect repair

**FIGURE 2:**
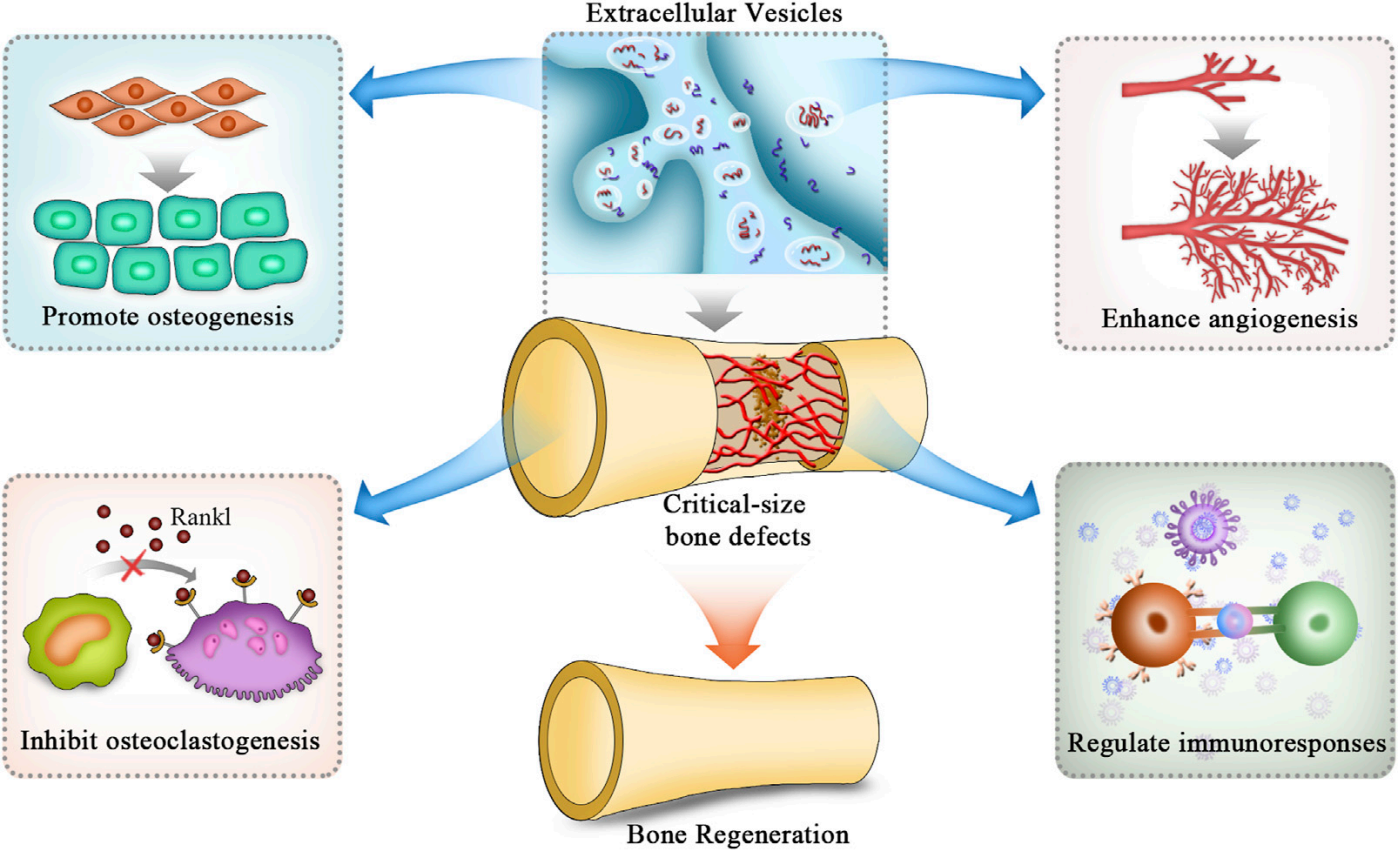
Mechanisms of EVs applied in BTE to repair critical-size bone defects. The mechanisms of EVs in repairing critical-size bone defects are mainly attributed to the promotion of osteogenic differentiation of stromal cells while inhibiting the differentiation of osteoclasts. In addition, they also promote angiogenesis and modulate inflammatory responses to provide a suitable environment for bone regeneration. EVs: extracellular vesicles.

In treating bone defects, EVs can regulate osteogenesis, angiogenesis, and immune responses, inhibit osteoclast activity, and finally promote repair processes by transporting their internal cargoes to target cells *via* downstream signalling pathways (Figure 2) (Gao et al., 2018). A large number of bioactive substances are contained in EVs, such as nucleic acids, proteins and lipids. Among these bioactive molecules, miRNAs are the most common cargoes (Toh et al., 2017). MiRNAs exert critical effects in cell-to-cell crosstalk and regulate bone tissue repair and regeneration by posttranscriptional modification. In addition to promoting osteogenesis and angiogenesis, EVs take part in regulating immune responses through their cargoes to suppress the activities of osteoclasts during the repair of critical-size bone defects (Li R. et al., 2022). Nevertheless, because EVs contain many miRNAs, these miRNAs may interact with each other to form a complex network. In addition to miRNAs, EVs also contain many proteins, lipids and other types of nucleic acids, such as long non-coding RNAs, circular RNAs and mRNAs. Thus, elucidating the intrinsic molecular mechanisms of EVs in regenerating bone tissues is necessary.

### 3.1 Extracellular vesicles promote critical-size bone defect repair by enhancing osteogenesis

The crux to repair critical-size bone defects is to enhance the activities of osteoblasts while inhibiting those of osteoclasts in the local microenvironment. Researchers have already confirmed that EVs can enhance the homing of endogenous stromal cells to defect sites, thereby shortening the time of bone formation and mineralization (Chew et al., 2019). In addition, EVs can bind matrix proteins, such as type I collagen and fibronectin, to induce the development and maturation of osteoblasts both *ex vivo* and *in vivo* (Narayanan et al., 2016). Some scholars used EVs derived from hBMMSCs with preosteogenic induction for RNA sequencing. The obtained results indicated that these EVs contained miR-196a, miR-27a and miR-206, which are important for osteogenic differentiation. Among those miRNAs, miR-196a has the potential to induce the expression of ALP, OCN, osteopontin and Runx2, leading to the formation of more bone tissues in critical-size calvarial defects (Qin et al., 2016b). Moreover, the expression of osteogenic-related miRNAs, such as miR-146a-5p, miR-503-5p, miR-483-3p and miR-129-5p, were upregulated, and the expression of anti-osteogenic-related miRNAs, such as miR-32-5p, miR-133a-3p and miR-204-5p, were downregulated. These miRNAs could activate the PI3K/Akt and MAPK signalling pathways to exert the biological effects of promoting bone mineralization in the local microenvironment (Zhai et al., 2020).

EVs promoting osteogenesis also involve several other signalling pathways, including BMP/Smad, Wnt/β-Catenin, tensin homologue/PI3K/Akt and HIPPO. These signalling pathways have been confirmed by many studies to play active roles in promoting the repair of critical-size bone defects (Cao et al., 2021). In addition to miRNAs, EVs can transport long non-coding RNAs to stromal cells at bone defect sites and indirectly promote bone mineralization by downregulating genes that inhibit osteogenesis (Yang et al., 2019). In addition to MSC-derived EVs, Qi et al. (2016) also demonstrated that human iPS-MSC-derived exosomes significantly promoted osteogenesis and angiogenesis through their internal miRNAs. The application of iPSC-MSC-derived exosomes together with β-TCP scaffolds promoted bone regeneration in critical-sized calvarial defects in an ovariectomized rat model. The results of Li et al. (2018) showed that in the critical-size bone defect microenvironment, in addition to activating the extracellular regulated protein kinase 1/2 pathways, hADSC-derived exosomes could also promote the expression of vascular endothelial growth factor (VEGF) in MSCs through various miRNAs, thus indirectly creating a good microenvironment for bone tissue regeneration by promoting angiogenesis.

Other researchers analysed the proteomics of osteoblast-derived EVs. The obtained results showed that up to 786 proteins in EVs were involved in osteogenic-related signalling pathways, such as mammalian target of rapamycin and eukaryotic initiation factor-2 (Ge et al., 2015). Similar studies have focused on EVs derived from mouse MC3T3 cells. Proteomic analysis found that among the 1,536 proteins, 172 were closely related to bone development and functions (Ge et al., 2017). Therefore, EVs may improve the differentiation ability of stromal cells or osteoblast precursor cells by transporting cargoes such as miRNAs and proteins in the bone defect microenvironment and enhancing the repair of critical-size bone defects.

### 3.2 Extracellular vesicles promote critical-size bone defect repair by suppressing osteoclastogenesis

Under normal circumstances, the regeneration processes of critical-size bone defects involve the imbalance of bone resorption and bone formation; that is, the activity of osteoblasts needs to be more dynamic than that of osteoclasts (Wu et al., 2010). Therefore, understanding the mechanisms by which EVs regulate osteoclast differentiation and formation will also provide new concepts for treating critical-size bone defects. EVs in the BTE can regulate the bone marrow microenvironment and inhibit osteoclast activities through their internal proteins and miRNAs. Prostate cancer cell-derived exosomes could downregulate miR-214 in osteoclasts and block NF-κB signalling, therefore inhibiting osteoclast differentiation (Duan et al., 2019). In addition, these exosomes could also inhibit osteoclast proliferation and differentiation by reducing the expression of osteoclast markers such as tartrate-resistant acid phosphatase, cathepsin K and matrix metalloproteinase-9, ultimately regulating bone formation (Karlsson et al., 2016). ADSC-derived exosomes not only reduce the mRNA and protein expression of RANKL but also reduce the ratio of RANKL/osteoprotegerin, thereby inhibiting RANKL-involved osteoclast maturation (Ren et al., 2019). EVs derived from endothelial cells could effectively inhibit bone resorption at bone defect sites by reducing osteoclast activities (Song et al., 2019). In addition, osteoclast-derived EVs could act as mediators between osteoclasts and osteoblasts, regulating the osteogenic function of osteoblasts. Osteoclast exosomes are rich in miRNAs, which inhibit osteoblast activity. MiR-214-3p in osteoclast-derived exosomes could target osteoblasts, inhibiting osteoblast activities *ex vivo* and deteriorating bone formation *in vivo*, while *in vivo* injection of antagomir-214-3p could effectively reverse these negative effects (Li D. et al., 2016). Therefore, miR-214 or miR-214-3p in EVs not only serve as biological tags of bone mass reduction but also as therapeutic targets to promote bone regeneration. Researchers also found that RANKL could induce the expression of miR-23a-5p in osteoclast-derived exosomes. Because miR-23a-5p suppresses the expression of Runx2 and promotes Yes-associated protein-1-mediated anti-osteogenic signalling, RANKL may be a target protein that regulates EV-mediated osteoclastogenesis differentiation (Yang J. X. et al., 2020). In conclusion, EVs may regulate osteoclast activities, thus mediating the development of bone regeneration; and some specific miRNAs can be used as therapeutic targets for bone defect repair. However, compared with the functional study of EVs promoting osteogenesis, there are currently fewer studies on the effects of EVs on osteoclast differentiation. Therefore, it is necessary to conduct more in-depth and comprehensive research in the future.

### 3.3 Extracellular vesicles promote critical-size bone defect repair by enhancing angiogenesis

Bone contains a high density of vasculature. Numerous bone cells participate in maintaining normal functions of bone, while the biological activities of these bone cells rely on the ambient vasculature. There is increasing evidence that maintaining adequate local blood supply or maintaining continuously increased angiogenesis plays an important role in critical-size bone defect regeneration (Wang et al., 2017). Therefore, one of the basic strategies for promoting the repair of critical-size bone defects is to promote angiogenesis (Jia et al., 2016). Various studies have demonstrated that EVs enhance angiogenesis near tendon-bone junctions *in vivo* (Vizoso et al., 2017), which is shown by promoting the expression of angiogenic factors and tube formation and stimulating the proliferation and migration of endothelial cells (Zhang B. et al., 2021). It was also confirmed that endothelial progenitor cell-derived exosomes enhanced the proliferation, migration and angiogenesis of endothelial cells by delivering miR-126. Transduced miR-126 improved the vascular areas and thicknesses around the shin bones of rats and finally accelerated the bone repair process in the defect sites (Jia et al., 2019). Scholars also found that miR-21 in MSC-derived exosomes downregulates the expression of sprouty homologue 2 and promotes angiogenesis (Wu et al., 2022). It was reported that BMMSC-derived exosomes may independently activate the VEGF or Hippo signalling pathway by regulating cell-to-cell contact and activating cytoskeleton dynamics (Wang Z. et al., 2021). Another study performed by Takeuchi et al. (2019) showed that MSC-derived EVs enhanced tissue and vascular development in parietal bone defects in Wistar rats by upregulating the mRNA expression of VEGF, angiopoietin 1 and 2 together with osteogenesis-related collagen I, ALP, OCN and osteopontin. Zhang et al. (2015) found that hiPSC-derived exosomes could directly promote angiogenesis and collagen synthesis. Similar studies have also demonstrated that EVs derived from MSCs could upregulate the expression of functional angiogenesis molecules and enhance the migration of hUVECs, exhibiting a larger area of angiogenic tube formation (Zhang et al., 2020). A rat model of femoral fracture demonstrated that transplanting MSC-derived EVs to the defect sites could significantly enhance angiogenesis and osteogenesis, leading to better bone tissue regeneration. Other researchers found that exosomes from apical papilla stem cells promoted angiogenesis through miR-126-5p, which was indirectly manifested by increased expression of VEGF and angiopoietin-1 (Jing et al., 2022). Scholars have also demonstrated that hypoxia pretreatment can regulate the expression of bioactive substances in exosomes, thus promoting angiogenesis in different microenvironments. After hypoxia preconditioning, the expression of miR-126 in MSC-derived exosomes was upregulated, which further promoted angiogenesis, proliferation and migration of stromal cells, thus enhancing the process of fracture healing (Liu et al., 2020). In addition to hypoxia, the proangiogenic ability of exosome components is also enhanced by certain biomaterials. A biomaterial containing lithium induced miR-130a expression in exosomes, activated tensin homologue/Akt signalling pathways, and finally promoted angiogenesis in the bone defect microenvironment (Liu et al., 2019). In conclusion, by selecting appropriate EVs and regulating the expression of bioactive substances within them, EVs may be promising candidates for treating critical-size bone defects as proangiogenic vesicles.

### 3.4 Extracellular vesicles promote critical-size bone defect repair by regulating immune responses

The healing processes of bone defects are often accompanied by local inflammatory responses and imbalanced immune reactions. These changes will always result in unsuccessful scaffold implantation in bone defect treatments (Guder et al., 2020). Large numbers of inflammatory and immune cells are present in the microenvironment of bone defect sites. Mild inflammation-related responses are critical for bone defect regeneration (Lin et al., 2021). The functions of EVs in immune response regulation are diverse. EVs not only participate in the processes of anti-inflammatory responses, such as in collagen-induced arthritis mice (Cosenza et al., 2018) and antigen-induced synovitis pigs (Casado et al., 2017) but also modulate beneficial regenerative immune phenotypes, which are important in tissue repair as well as calcification (Lindenbergh and Stoorvogel, 2018). In addition, EVs can regulate the functions of T-cell, B cells, macrophages and other immune cells directly by membrane fusion or indirectly by transporting bioactive cargoes (Qian et al., 2021). It has already been demonstrated that MSC-derived exosomes are effective in suppressing immune responses by attenuating the proliferation of T-cell and B cells. Moreover, MSC-derived exosomes can also suppress the secretion of the proinflammatory cytokines tumour necrosis factor-α and IL-1β, together with increasing the excretion of the anti-inflammatory cytokine TGF-β *in vitro* (Chen et al., 2016), thus maintaining a favourable immune balance that was crucial for bone healing. Macrophages are part of innate immunity. They participate in removing pathogens and modulating inflammatory responses in the human body (Bozec and Soulat, 2017). Macrophage M2 polarization can enhance angiogenesis and bone tissue regeneration (Wasnik et al., 2018). EVs loaded into scaffolds could promote macrophage M2 polarization and suppress the inflammatory responses in the local microenvironment, as manifested by decreased mRNA and protein expression of the proinflammatory factors IL-6 and tumour necrosis factor-α (Guan et al., 2021). The inhibited inflammatory responses would indirectly boost vascularization, therefore facilitating osteoblast function and bone tissue mineralization and finally leading to favourable new bone tissue formation (Kim et al., 2021). This EV-mediated transplantation system has been recognized as a novel alternative for treating bone defects. Further mechanistic studies showed that the NF-κB pathway may play an important role in this process (Fan et al., 2021). To further confirm whether the bioactive molecules of MSC-derived exosomes play a key role in regulating immune responses, Li R. et al. (2022) analysed the role of miR-451a, which is highly expressed in ADSC-derived exosomes, in repairing rat skull defects. By transfecting miR-451a analogues and inhibitors into macrophages, it was found that miR-451a could directly regulate the mRNA expression of macrophage migration inhibitory factor by specifically binding to its 3′UTR, thereby promoting the polarization of the macrophage phenotype from M1 to M2. Moreover, the results of other studies also found that UVEC-derived exosomes, in which programmed cell death ligand 1 was overexpressed, could bind to programmed cell death-1 on the surface of T-cell, thereby inhibiting its activation. As a result, the osteogenic differentiation of MSCs was significantly enhanced after inhibiting the local hyperactivated inflammatory responses (Lin et al., 2021). Therefore, EVs are critical modulators of immune responses for regenerating new bone tissues.

In summary, the roles of EVs in repairing critical-size bone defects are attributed to 1) promoting osteogenic differentiation of stromal cells while inhibiting the differentiation of osteoclasts; 2) enhancing angiogenesis to provide a suitable environment for bone regeneration; and 3) modulating inflammatory responses to maintain a favourable immune microenvironment (Figure 2). Thus, EVs would be an ideal cell-free therapeutic component to treat critical-size bone defects. As we have reviewed, mechanistic studies of EV application in BTE are mainly performed from the aspect of internal cargoes of miRNAs and proteins. More studies focused on these are summarized in Table 2.

**TABLE 2:** Therapeutic roles and possible mechanisms of certain miRNAs and proteins of EVs in critical-size bone defects repair.

Cargoes of EVs	Parent cells	Nanovesicles	Therapeutic roles and possible mechanisms	References
miR-196a	BMMSCs	EVs	Stimulating bone growth in critical-sized calvarial bone defects through enhancing osteoblast activity and the expression of osteogenic genes	Qin et al. (2016b)
miR-218, miR-92a, miR-199b	BMMSCs	Exosomes	Promoting osteogenic differentiation of BMMSCs	Xu et al. (2014)
miR-26a-5p	BMMSCs	Exosomes	Inhibiting the damage of synovial fibroblasts by targeting prostaglandin-endoperoxide synthase 2	Jin et al. (2020)
miR-192-5p	BMMSCs	Exosomes	Inhibiting local inflammatory responses by regulating the expression of proinflammatory factors	Zheng et al. (2020)
miR-328-3p	BMMSCs	Apoptotic bodies	Maintaing MSCs homeostasis and ameliorating osteopenia through inhibiting Axin1 and thereby activate the Wnt/β-catenin pathway	Liu et al. (2018)
miR-34	BMMSCs, ADSCs	Exosomes	Promoting proliferation and osteogenic differentiation of BMMSCs	Baglio et al. (2015)
miR-375	ADSCs	Exosomes	Promoting bone regeneration by binding to insulin growth factor binding protein-3	Chen et al. (2019)
miR-451a	ADSCs	Exosomes	Regulating bone immune metabolism and further promoting bone healing through targeting macrophage migration inhibitory factor	Li et al. (2022b)
miR-503-3p	Osteoblast	Exosomes	Inhibiting the osteoclast differentiation through downregulating the expression of heparanase	Wang et al. (2021a)
miR-1192, miR-680, miR-302a	Pre-osteoblast Osteoblast	Exosomes	Promoting osteoblastic differentiation by inhibiting Axin1 expression and increasing β-catenin expression to activate the Wnt signaling	Cui et al. (2016)
miR-8485	Chondrocyte	Exosomes	Regulating the Wnt/β-catenin pathways to promote chondrogenic differentiation of BMMSCs	Li et al. (2020c)
miR-221-3p	Chondrogenic progenitor cells	EVs	Stimulating chondrocyte proliferation and migration	Wang et al. (2020c)
miR-214	Endothelial cells	Exosomes	Stimulating angiogenesis through silencing the ataxia telangiectasia mutated gene in neighboring target cells	van Balkom et al. (2013) Duan et al. (2019)
	Prostate cancer cell		Inhibiting osteoclast differentiation by blocking the NF-κB signaling pathway	
miR-155	Endothelial cells	Exosomes	Indirectly inhibiting osteoclast activity by interacting with macrophages	Song et al. (2019)
miR-126	Endothelial progenitor cell	Exosomes	Promoting angiogenesis *via* Raf/ERK signaling pathway	Jia et al. (2019)
miR-335	DC	Exosomes	Enhancing bone regeneration by promoting the proliferation and osteogenic differentiation of BMMSCs through inhibiting Hippo signaling targeting large tongue suppressor kinase 1	Cao et al. (2021)
miR-5106	M2 macrophages	Exosomes	Inducing osteoblast differentiation by regulating the expression of salt-inducible kinase 2/3	Xiong et al. (2020)
miR-23a	Nasopharyngeal carcinoma cells	Exosomes	Mediating angiogenesis by targeting The testis- specific protein 10	Bao et al. (2018)
miR-135b	hypoxia-resistant multiple myeloma cells	Exosomes	Enhancing endothelial tube formation under hypoxia *via* the HIF signaling pathway	Umezu et al. (2014)
Ring finger protein 146	BMMSCs	Apoptotic bodies	Maintaing MSCs homeostasis and ameliorating osteopenia through inhibiting Axin1 and activating the Wnt/β-catenin pathway	Liu et al. (2018)
Membrane cofactor protein-1/3, Stromal cell derived factor-1	BMMSCs	Exosomes	Promoting cell proliferation and MSCs recruitment to injury sites	Furuta et al. (2016) Ando et al. (2014)
BMP2	BMMSCs	Exosomes	Enhancing fracture healing by promoting osteogenesis and angiogenesis through activating BMP-2/Smad1/Runx2 and the HIF-1α/VEGF signaling pathways	Zhang et al. (2020)
HIF-1α, VEGF	hUCMSCs	Exosomes	Enhancing angiogenesis and bone healing processes by promoting vascular endothelial proliferation, migration and tube formation	Zhang et al. (2019)
C-Type lectin domain family 11-Member A	hUCMSCs	EVs	Preventing bone loss and maintaining bone strength by enhancing bone formation, reducing marrow fat accumulation and decreasing bone resorption through shifting from adipogenic to osteogenic differentiation of BMMSCs and inhibiting osteoclast formation	Hu et al. (2020)
Matrix metalloproteinase 2	Osteoblasts	Exosomes	Promoting the angiogenesis of endothelial cells through VEGF signaling pathway	Tang et al. (2019)
Annexin	Osteoblasts	EVs	Inducing osteogenesis, calcium channeling by activating Wnt proteins	Xiao et al. (2007)
Platelet derived growth factor	Osteoclasts	Apoptotic bodies	Inducing endothelial progenitor cell Differentiation in a bone defect model	Ma et al. (2021)
TGF-β1, IL-10	DC	Exosomes	Enhancing the recruitment of regulatory T-cell in inflammatory responses and inhibiting bone loss caused by osteoclasts	Elashiry et al. (2020)
Insulin growth factor-1	Macrophages	Microvesicles	Redirecting epithelial cells towards internalizing microvesicles	Blander (2017)

ADSCs: adipose derived stem cells; BMMSCs: bone marrow mesenchymal stem cells; BMP2: bone morphogenetic protein 2; DC: dendritic cell; EVs: extracellular vesicles; HIF-1α: hypoxia inducible factor-1α; IL: interleukin; MSCs: mesenchymal stem cells; NF-κB: nuclear factor-κB; Runx2: runt-related transcription factor 2; Smad: mothers against decapentaplegic homolog; TGF: transforming growth factor; UCMSCs: umbilical cord mesenchymal stem cells; VEGF: vascular endothelial growth factor.

## 4 Application of engineering modified extracellular vesicles in bone tissue engineering

Although natural EVs exert diverse functions in regenerative medicine through multiple biological mechanisms, their limitations in repairing critical-size bone defects have gradually been recognized. Specifically, the targeting effect of natural EVs is poor. Most of the mature EVs delivered to bone defect sites will enter the circulatory system and cannot all accurately act on the target cells. In addition, not all EVs isolated from parent cells have therapeutic effects; thus, the number of effective natural EVs is far from sufficient. As a result, the bioactive molecules that are needed in different pathological circumstances cannot reach a therapeutic concentration. Although EVs are processed and assembled in parent cells and inherit similar biological characteristics to them, the bioactive molecules in natural EVs vary greatly, which will bring unstable efficacy in repairing critical-size bone defects. Therefore, engineering-modified EVs have been investigated to accelerate the bone regeneration process by applying multiple techniques that fall into two main categories in most circumstances: endogenous engineering strategies and exogenous engineering strategies. Advantages and disadvantages of some commonly used techniques are summarized in Table 3. Applications of engineered modified EVs can enhance therapeutic efficiency and more accurate targeting effects over natural EVs (Arenaccio et al., 2019).

**TABLE 3:** Commonly applied techniques for engineering modified EVs preparation.

Techniques	Operation approaches	Advantages	Disadvantages	References
Genetic engineering	Transfecting parent cells by plasmid or virus carrying target genes	Significantly increase the therapeutic effects of EVs through modifying their loading cargoes	High technical request and long preparation time	Stickney et al. (2016)
Preconditioning	Co-incubating parent cells or EVs with drugs or reagents	Easy to operate and the membranes of parent cells or EVs keep intact	Cargoes loading efficiency is relatively low and the drugs or reagents may have toxicity to parent cells or EVs	Pascucci et al. (2014)
Electroporation	Exerting electric field to parent cells or EVs suspended in conducting solution, exogenous siRNA or miRNA would be easy to load through the perforated membrane	Both exogenous macromoleculars and hydrophilic small molecular compounds could be loaded	The integrity of the membrane is destroyed, leading to unstable biological characteristics of parent cells or EVs	Johnsen et al. (2016)
Mechanical extrusion	Mixing parent cells or EVs with drugs or reagents and then process by extrusion	Easy to operate and more exogenous substances could be loaded	The integrity of the membrane is destroyed, leading to unstable biological characteristics of parent cells or EVs	Fuhrmann et al. (2015) Wan et al. (2018)
Sonication	Mixing EVs with drugs or reagents and then process by sonication treatment	More exogenous substances could be loaded with higher loading efficiency	The integrity of the EVs membrane is destroyed with uncontrollable loading capacity of target drugs	Kim et al. (2016)
Freezing/thawing	Rapid freezing mixed EVs and drugs or reagents and then thaw at room temperature	Easy to operate and the membrane of EVs keeps intact	Cargoes loading efficiency is relatively low and the biological activities of EVs or drugs might change	Sato et al. (2016)
Chemical conjugation	Conjugating target moleculars to the surface of EVs directly through covalent bond	Increasing the EVs targeting characteristic while keeping the morphology and structure of the membrane intact	Difficult to operate and only molecular with specific functional group could be loaded	Tian et al. (2018) Zhao et al. (2019)

EV: extracellular vesicle.

### 4.1 Endogenous engineering modified extracellular vesicles

In the previous sections, the authors summarized the biological characteristics of EVs derived from different parent cells and their applications in BTE to repair critical-size bone defects. Studies have shown that except for selecting appropriate types of parent cells, modification of the parent cells before EV isolation through genetic strategies, preconditioning treatment and physical manipulations could also modify the biological characteristics of EVs (Jafari et al., 2020). These approaches are categorized as endogenous engineering modifications*.*

#### 4.1.1 Genetic engineering strategies

Genetically modifying the natural procedures of protein synthesis of parent cells can endow the modified EVs with membrane modification and more desirable functions, such as precise targeting and individualized therapeutic performance, by regulating some targeted biomolecules. Compared with natural EVs, specific ligands can be added to the EV membrane to improve its targeting effects through genetic engineering (Hu et al., 2021). After transfection with the *RUNX2* gene, the osteogenic differentiation of hBMMSCs was potentiated. Meanwhile, the differentiation induction capabilities of EVs derived from this gene-transfected cell were also enhanced *in vitro* (Martins et al., 2016). In a similar study, the expression of HIF-1α in parental MSCs was upregulated. The EVs derived from these cells exhibited better regenerative characteristics. The *in vitro* results indicated that EVs from HIF-1α-upregulated MSCs could better enhance matrix mineralization, ALP activity and osteogenesis-related gene expression in BMMSCs and promote the migration, proliferation and tube formation of HUVECs. Meanwhile, *in vivo* results indicated better bone tissue regeneration and neovascularization; therefore, the trabecula bone healing time was significantly shortened in rabbit necrotic femoral heads (Li et al., 2017). Another study also upregulated HIF-1α expression in BMMSCs. Their derived exosomes were loaded into a β-TCP scaffold to repair critical-size bone defects in rats. The obtained results also confirmed that compared with applying normal BMMSC-derived exosomes, exosomes derived from HIF-1α-upregulated BMMSCs considerably accelerated the regeneration of new bone tissues with favourable stromal cell neovascularization differentiation (Ying et al., 2020). Other researchers have tried to activate the endogenous BMP signalling pathway by downregulating the natural BMP antagonist noggin in the parent cells of EVs (Fan et al., 2016). They observed EVs obtained from noggin knockdown MSCs and found that a large number of endogenous aggregations of osteogenic-related molecules appeared in EVs. When these modified EVs were loaded into injectable ethylene glycol methacrylate chitosan hydrogels and transplanted into mouse critical-size calvarial defects, more desired bone regeneration could be observed (Fan et al., 2020).

#### 4.1.2 Preconditioning treatment of parent cells

Preconditioning treatment of parent cells through conditioned culturing medium could improve the biological activities of EVs derived from them. It is an effective strategy to boost EV-mediated BTE in regenerating bone tissues. Preconditioning treatment can directly modify the DNA or RNA of the parent cells to obtain needed EVs rich in target cargoes. Narayanan et al. (2018) found that exosomes derived from MSCs that were precultured in osteogenic induction medium showed better capabilities of inducing osteogenic differentiation of other MSCs *in vitro* and of promoting neovascularization in 3D cultures *in vivo*. Liu A. et al. (2021) compared EVs derived from different types of stem cell sources and culturing media. The obtained results suggested that exosomes from rBMMSCs preconditioned with osteogenic induction medium exhibited the most desirable osteogenesis characteristics, and the ALP activity increased by more than 2 times compared with the blank control. In-depth bioinformatics analysis further confirmed that preconditioning rBMMSCs with osteogenic induction medium could upregulate the expression of multiple osteogenic-related miRNAs in their derived EVs such as let-7a-5p, let-7c-5p, miR-328a-5p and miR-31a-59. Mesoporous bioactive glass scaffold materials loaded with these modified exosomes could effectively repair rat skull defects *in vivo*. Another study found that exosomes derived from hBMMSCs preconditioned with dimethyloxaloylglycine, which stabilizes HIF-1α at a low concentration, could improve bone healing by boosting angiogenesis in critical-sized calvarial defects in rats (Liang et al., 2019). Moreover, Huang C. C. et al. (2020) demonstrated that in a rat skull critical-size defect model, compared with natural hBMMSC-derived EVs, BMP2 preconditioned hBMMSC-derived EVs had significantly enhanced bone regeneration ability. Other scholars used BMP2 to precondition mouse macrophages (RAW 264.7) and observed the osteogenic potential of their exosomes in bone regeneration applications (Wei F. et al., 2019). The obtained results showed that the preconditioned macrophage-derived EVs enhanced the osteogenic differentiation of BMMSCs. These results demonstrated that preconditioned parent cell-derived EVs could be better applied in bone regeneration.

Another widely applied preconditioning treatment of parent cells is a hypoxic environment. MSCs, which were preconditioned in a hypoxic environment, promoted angiogenesis capability. In addition, EVs derived from these cells also had better angiogenic characteristics and led to enhanced osteogenic capability. The *in vitro* experiments showed that these modified EVs could promote the proliferation and migration of HUVECs and tube formation. Due to the favourable angiogenic microenvironment, osteogenic differentiation of BMMSCs at the bone defect site was also enhanced, which was manifested by increased gene expression of serum OCN and ALP (Li et al., 2017). The *in vivo* experiments were performed using the steroid-induced avascular necrosis model in the femoral head. The obtained results indicated that the femoral head transplanted into EVs derived from hypoxia-preconditioned cells had higher vascular density and denser trabecular bone tissue formation (Li et al., 2017). The results of Sakaguchi et al. (2017) were similar. Researchers also found that exosomes derived from BMMSCs in a hypoxic environment could enhance the formation of tubular structures by HUVECs. Compared with natural exosomes, *in vivo* applications of modified EVs showed more favourable new bone formation.

#### 4.1.3 Physical manipulation approaches

Physical manipulation could change the amount of EVs released from parent cells together with the type and amount of therapeutic cargoes in EVs. Generally, physical manipulation is performed by directly exerting physical forces on the parent cells by electroporation and extrusion (Richter et al., 2021), thereby optimizing EVs derived from them. Cui et al. (2022) reported bioinspired nanovesicles prepared from human iPSC-derived endothelial cells under hypoxia culture through an extrusion approach. Abundant membrane C-X-C motif chemokine receptor 4 conferred these nanovesicles bone-targeting ability and the endothelial homology facilitated the bone marrow endothelial cells tropism. Due to their unique endogenous miRNA cargoes, these nanovesicles re-educated bone marrow endothelial cells to secret cytokines favoring osteogenesis and anti-inflammation, leading to a better bone homeostasis. Researchers also used hydroxyapatite ceramics containing magnetic nanoparticles as a scaffold material to simultaneously load osteoblasts and osteoclasts. They found that magnetic nanoparticles could change the internal cargoes of osteoclast-derived exosomes, resulting in a decreased amount of osteoclast-derived exosomes absorbed by osteoblasts, thus weakening the negative regulatory effect of osteoclasts on osteoblasts. Osteoblasts showed enhanced proliferation and differentiation capabilities (Zhu et al., 2020). Further studies found that changes in the cargoes of exosomes derived from osteoclasts were mainly reflected in the magnetic nanoparticle-mediated upregulation of Rho kinase and the downregulation of ubiquitination and reactive oxygen species.

### 4.2 Exogenous engineering modified extracellular vesicles

To summarize briefly, exogenous engineering of EVs involves functional modification after their isolation, directly loading specific external cargoes into nanoscale EVs to deliver bioactive molecules with therapeutic effects to the local microenvironment, thereby promoting critical-size bone defect repair (Zha et al., 2021). Currently, engineered EVs can be prepared by incubation, electroporation, sonication, mechanical extrusion, recurrent freezing and thawing, and direct EV surface modification by applying covalent or non-covalent interactions (Liao et al., 2019). Using these techniques, the modified EVs would load a desired amount of therapeutic biomolecules, including small molecular drugs, nucleic acids, and proteins either inside or on the surface. In addition, these modified EVs specifically bind antibodies through covalent bonds on lipid membranes and ligands.

Liang et al. (2020) added a plasmid encoding BMP2 to EVs derived from rat MSCs to potentiate the bone inducibility of demineralized bone matrix. The specific method was to coat the membrane of EVs with polyethyleneimine, a cationic polymer usually applied to coat negatively charged DNA, and then add the plasmid BMP2 through a layer-by-layer self-assembly method. This easy technique constructed 100–1,000 nm diameter EV-polyethyleneimine/plasmid BMP2 complexes. Compared with the control group, EV complexes showed upregulated BMP2 gene transfection efficiency and lower toxicity to target cells *in vitro*. In addition, demineralized bone matrix loaded with these novel complexes exhibited increased bone regeneration induction capability after being implanted subcutaneously in rat and rabbit femoral head bone defect models.

VEGF plays a critical role in angiogenesis and BTE for bone tissue regeneration (Grosso et al., 2017). However, as a protein, VEGF has many problems in its direct delivery, including rapid degradation, uncontrolled release and unstable biological activities (Hu and Olsen, 2016). Zha et al. (2020) designed a matrix that could locally release VEGF plasmids for bone defect repair. They used EVs derived from rBMMSCs to transport the plasmid VEGF, which was equivalent to providing a protective layer for VEGF, thus avoiding the problems mentioned above, such as rapid degradation. Researchers first used the electroporation technique to temporarily create holes on the surface of EVs by an electric pulse to increase membrane permeability. After that, VEGF plasmids were loaded into EVs. The modified EVs successfully promoted vascularization and osteogenesis in rat skull defects. In addition, it could also effectively achieve vascularized bone regeneration in segmental bone defects (Zha et al., 2021).

Cui et al. (2021) developed an exosome delivery system based on exosomes secreted by iPSC-MSC. The engineered exosomes collaborated with the loaded siRNA of the *SHN3* gene to enhance their therapeutic effects. Modification of a bone-targeting peptide endowed the engineered exosomes an ability to deliver siRNA to osteoblasts specifically. Silencing of the osteoblastic *SHN3* gene enhanced osteogenic differentiation, and inhibited osteoclast formation. Furthermore, *SHN3* gene silencing could facilitated vascularization, especially formation of type H vessels. This study demonstrated that exogenous engineered exosomes could serve as a promising therapy vesicle to kill three birds with one stone. Luo et al. (2019) also provided a promising method to enhance the ability of exosomes to target specific tissues. In their study, the surface of exosomes from bone marrow stromal cell was conjugated with a BMSC-specific aptamer, which delivers exosomes into BMSCs within bone marrow. Intravenous injection of the exosome-aptamer complex enhances bone mass in osteoporosis mice and accelerates bone regeneration in a femur fracture mouse model.

## 5 Future perspectives and challenges

**FIGURE 3:**
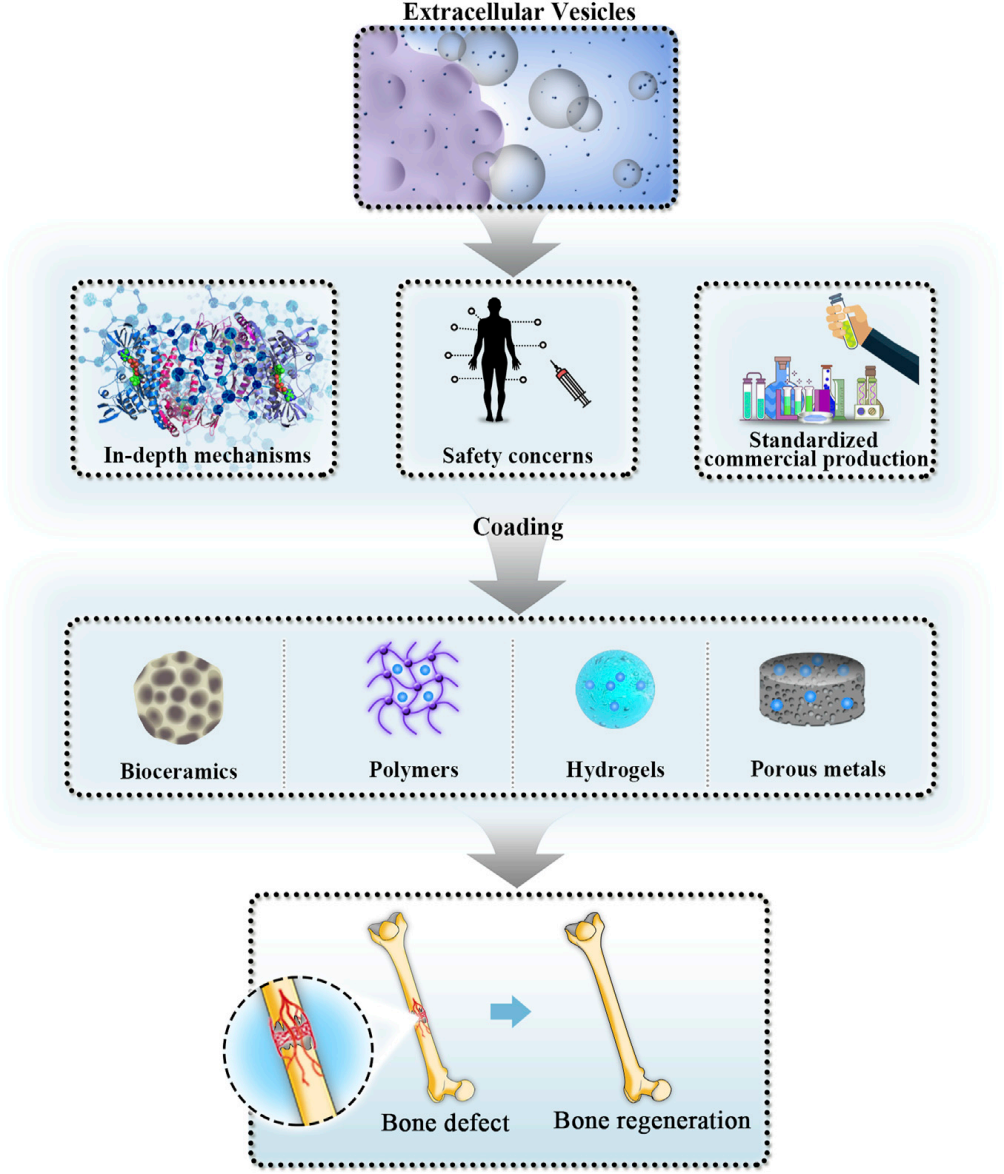
Future perspectives and challenges of EV clinical application in BTE. EVs have shown exciting application prospects in repairing critical-size bone defects. In the near future, scholars will focus on studying in-depth mechanisms, solving safety concerns and promoting standardized commercial production of EVs applied in BTE. With further development of bioactive scaffold materials, cell-free regenerative medicine in BTE will soon be widely used. BTE: bone tissue engineering; EVs: extracellular vesicles.

With meticulous and thorough research exploring the biogenesis and biofunctions of EVs, natural and engineered modified EVs that possess various promising advantages, such as biological functions similar to those of parent cells, low immunorejection, desirable biocompatibility, and the capability to deliver individualized drugs, have shown exciting application prospects in diagnosing and treating diverse pathological conditions (Nagelkerke et al., 2021). The first phase I clinical trial on EVs derived from DCs was conducted for patients with malignant melanoma, and the obtained results indicated that their clinical application was safe (Escudier et al., 2005). Although this clinical safety evaluation trial started as early as 2005, further clinical studies focusing on the evaluation of the safety and effectiveness of EVs applied in tissue repair and regeneration are still in progress mainly in phases I and II. As an important part of BTE development, the progress of EVs in the future will largely determine the progress of BTE-led critical-size bone defect repair and regeneration. Certain achievements have been made in preclinical trials of EV-mediated nanotherapies, and the obtained results showed ideal effects in bone fracture healing (Zhang et al., 2019). However, scholars believe that there are still some unsolved theoretical challenges and important practical problems that should be addressed during future and wider clinical utilization (Figure 3). Nevertheless, challenges and difficulties could not hinder EVs from being a promising alternative for MSC-based therapy in bone regenerative medicine.

### 5.1 More in-depth mechanisms of extracellular vesicle-mediated bone defect regeneration

Although extensive studies have focused on various therapeutic mechanisms of natural or engineered modified EVs for different diseases, such as cancer and inflammatory diseases (Karlsson et al., 2016; Cosenza et al., 2018), the potential roles of specific functional units from EVs in bone defect regeneration have not been completely explained. In this review, the authors summarized some of the most widely raised clinical mechanisms of EVs as regenerative agents, such as immune regulation, as well as regulation of cell osteogenesis or angiogenesis. However, the detailed therapeutic mechanisms or related downstream targeted signalling cascades largely remain unclear and need to be further explored. These studies are of basic critical importance for scholars to comprehend the therapeutic effects of EVs in regenerative medicine and the biomedical applications of EVs.

Future studies at this stage will mainly focus on exploring the mechanisms of inducing bone regeneration and manufacturing more ideal scaffold materials. Theoretically, EVs regulate bone defect repair and regeneration through the interaction of their multiple internal cargoes (Arenaccio et al., 2019). However, most of the currently available studies focused on the roles of miRNAs in EVs, which indicated that the mechanisms of EVs in bone regeneration are far from being fully explored. It is also necessary to further elucidate the different types of effective active factors of EVs that promote osteogenic differentiation, angiogenesis, inflammation regulation and osteoclast inhibition. In addition, as previously mentioned, the *in vivo* application of EVs cannot be separated from the scaffold materials. In current studies, based on the combined applications of different scaffold materials, scholars have developed a variety of innovative methods to improve the delivery efficiency of EVs (Liao et al., 2019). However, problems such as ineffective delivery and lack of long-term retention of EVs still exist (Riau et al., 2019). In the future, with the development of innovative scaffold materials and standardization of the porosity value or size of biocompatible 3D porous scaffolds for bone defects, efficient transportation, sustained release and long-term storage of EVs will be achieved.

Furthermore, deeper investigations of *in vivo* bioeffects of EVs, such as distribution or bioelimination in blood circulation or in diverse pathological microenvironments, will also provide a deeper understanding of the pharmacokinetic and pharmacodynamic performances of EVs. This broader understanding will be beneficial to the proposal of EV-mediated targeted nanotherapeutic strategies.

### 5.2 Potential safety concerns of extracellular vesicles in clinical applications

As reviewed earlier, EVs have more favourable biocompatibility and enhanced biosafety over conventional stem cell therapy; however, the whole components of EVs, including some other unknown bioactive molecules, are still not completely clear (Phan et al., 2018). Although the Extracellular RNA Communication Consortium (Das et al., 2019) and the International Society for Extracellular Vesicles (Théry et al., 2018) have published guidelines to recommend researchers in standardizing the isolation and characterization procedures of EVs, the applications of EVs in the clinic would still lead to some unpredictable or undesirable biological effects. On the other hand, the heterogeneous constituents of EVs may lead to side effects that accumulate in non-target organs (Phan et al., 2018). Thus, more efforts to clarify the compositions of EVs are needed to avoid possible side effects during clinical applications in the human body.

Currently, the optimal therapeutic dose of EVs is still uncertain; thus, there would also be potential side effects after application. More preclinical studies are needed, especially on applying EVs in bone defect repair in large animals, to provide more safety references and a basis for the clinical promotion of EVs in later stages.

### 5.3 Standardized and commercialized production of extracellular vesicles

Effective isolation of EVs is the basis for their clinical application. From this point of view, standardized purification, preparation, production and preservation are essential. Increasing evidence indicates that EVs from different types of parent cells carry distinct bioactive molecules that play different biological functions in bone remodelling and regeneration (Tian et al., 2018). Even EVs derived from the same parent cell still have diverse sizes, component cargoes, proteins, and other biophysics or biochemical properties (Yan et al., 2020). Thus, only by solving the standardizing issues can we realize a safer clinical application of EVs.

Currently, differential ultracentrifugation and sequential ultrafiltration are the most commonly employed approaches to isolate EVs (Richter et al., 2021). However, these techniques have certain disadvantages, including being time-consuming, having low EV purity and output, and having unfavourable reproducibility (Théry et al., 2018). Commercialized isolation kits for EVs are now available but have not overcome the low efficiency limitation and are expensive. Therefore, the development of a standardized and time-saving isolation technique to harvest high-purity EVs at low costs would be a cornerstone to eventually achieve commercialized clinical application of EVs.

Accordingly, technology to accurately regulate the expression of bioactive molecules in EVs is crucial to achieve more desirable tissue repair. Therefore, we will further explore the engineering strategies of EVs through gene modification, specific preconditioning treatment, external gene/protein loading or combination to prepare EVs with enhanced bone induction ability. In addition to engineering modification of EVs, improving the loading efficiency of internal cargoes without interfering with the natural lipid and protein compositions of EVs is also an important issue that cannot be ignored.

## 6 Conclusion

Critical-size bone defects have become a serious threat to populations at both the individual and societal levels. The progress of stem cell therapy has led to promising results for remodelling and regeneration of bone tissues. However, stem cell therapy has some disadvantages that restrict its wider clinical applications. These disadvantages, together with problems caused by storage and transport, further limit the application of MSCs in repairing critical-size bone defects. An increasing amount of convincing evidence has demonstrated that most beneficial functions of stem cells are attributed to EVs, which gives rise to a new paradigm in which EVs secreted from both stem cells and other cells promote bone regeneration by modulating crosstalk between EVs and resident bone cells or the surrounding microenvironment. Although the utilization of EVs as bioactive nanotherapeutics in regenerative medicine has just started from the preliminary stage, the results of preclinical tests showed that applications of EVs would overcome limitations of stem cell-based therapies and possible side effects associated with the administration of stem cells. In addition, some unique advantages of EVs, including low immune rejection, feasibility to modify membrane constituents, ability to pass the blood‒brain barrier, convenient isolation, and lack of tumorigenicity, make the application of EVs a promising choice in biomedical treatment for critical-size bone defects.

Accumulated findings suggest that EV-mediated therapy may be an effective alternative to regulate the bone metabolism microenvironment. Here, the authors reviewed the molecular mechanisms of the effects of EVs on osteogenesis, osteoclast differentiation, angiogenesis, and immune reactions. It is well-recognized that EVs derived from different sources have different internal cargoes, such as inflammatory cytokines, signalling molecules, mRNAs, miRNAs and lncRNAs. The diverse capabilities of EVs come from different internal cargoes. Currently, scholars mainly focus on miRNAs, which play a critical role in EV-mediated critical-size bone defect regeneration. However, researchers had no information about critical proteins and other RNAs involved in the interactions between different EVs and EV-target cells, especially in distinct pathological circumstances. These problems must be of great interest to future preclinical and clinical studies aiming to explore the underlying therapeutic mechanisms of EVs. In addition to mechanistic studies, further functional studies of EV-mediated therapy are also needed, as EVs extracted *ex vivo* do not exactly mirror those extracted *in vivo* when parent cells are exposed to specific diseases or injury-related conditions.

Efficient delivery, bioactivity maintenance, and controlled/sustained release will become key factors for the safe and effective application of EVs in critical-size bone defect repair. Before resolving these issues, the isolation and characterization techniques need to be standardized first, but the field has yet to achieve consensus. Although scholars in EV-mediated BTE face numerous challenges, with continued advances in understanding EV biology and therapeutic mechanisms, together with the rapid development of related disciplines, such as bioengineering, chemistry, material science, and nanotechnology, it is believed that EVs will stimulate further explorations and inspirations for tissue engineering and efficient nanotherapeutics in the near future, eventually bringing the spring of cell-free regenerative medicine for critical-size bone defect repair.

## Abbreviations

ADSCs, adipose-derived stem cells; ALP, alkaline phosphatase; BMMSCs, bone marrow mesenchymal stem cells; BMP2, bone morphogenetic protein 2; BTE, bone tissue engineering; DC, dendritic cell; EVs, extracellular vesicles; HIF-1α, hypoxia inducible factor-1α; IL, interleukin; iPS-MSCs, MSCs derived from induced pluripotent stem cells; MAPK, mitogen-activated protein kinase; MSCs, mesenchymal stem cells; NF-κB, nuclear factor-κB; OCN, osteocalcin; PI3K, phosphoinositide 3-kinase; PLGA, polylactic-co-glycolic acid; RANKL, receptor activator of nuclear factor-κB ligand; Runx2, runt-related transcription factor 2; SHEDs, stem cells from human exfoliated deciduous teeth; Smad4, mothers against decapentaplegic homologue 4; TCP, tricalcium phosphate; TGF, transforming growth factor; UCMSCs, umbilical cord mesenchymal stem cells; UVECs, umbilical vein endothelial cells; VEGF, vascular endothelial growth factor.
